# Supplementary material for: Association between mild cognitive impairment and lumbar degenerative disease in a Japanese community: A cross-sectional study
Source: PLoS One. 2021 Oct 19;16(10):e0258852. doi: 10.1371/journal.pone.0258852 (PMC8525772; doi:10.1371/journal.pone.0258852)
Supplement: Attachment — Submitted filename: Editor reviewer replies_revision.docx [file pone.0258852.s001.docx]

Editor and reviewer comments and replies

Comment 1 from editor

How about the use of analgesic drugs or other drugs such as pregabalin, anti-depressant, or benzodiazepines?

Reply 1 to editor

We thank the editor for providing advice on the current study, thereby improving the manuscript. The percentage of participants who used sleeping pills, analgesic drugs, and psychotropic drugs were 8.3% (28/336), 7.4% (25/336), and 1.5% (5/336). However, we did not have detailed drug information, such as the drug names, in this study. Analgesic drugs or other drugs would mask their symptoms, and we might underestimate cognitive impairment and/or lumbar degenerative disease. Thus, we have described the effect of the absence of drug information on the relationship between cognitive function and lumbar degenerative disease as a limitation of this study (page 22, lines 303-306).

Comment 2 from editor

Did the authors check MMSE just one time? Sometimes, MMSE score maybe changed.

Reply 2 to editor

Thank you for pointing out an important issue of our evaluations. We checked MMSE just one time in this study. As you mentioned, MMSE score may be changed by mental and physical conditions of individuals, and we have described this in the study limitations (page 22, line 306-307).

Comment 3 from editor

How about the depression scores in these subjects?

Reply 3 to editor

We appreciate the editor’s comment and agree that it is necessary to evaluate depression scores in this study. We have evaluated depression using The Center for Epidemiologic Studies Depression Scale and set a cut-off point at 16 points or greater. We have repeated the statistical analysis and revised the material and method section (page 8, lines 124-128), results, and tables (table 1 to 4).

Comment 4 from reviewer

The title needs to be rethought, as it is not entirely clear regarding the content of the paper.

Reply 4 to reviewer

Thank you for your valuable advice to improve the title of our study. We have changed the title to “Association between mild cognitive impairment and lumbar degenerative disease in a Japanese community: a cross-sectional study.”

Comment 5 from reviewer

Regarding the introduction, it would be positive to include a brief explanation about the "super-aging society".

Reply 5 to reviewer

Thank you for your valuable comment. “Super-aging society” means that the proportion of older adults whose age is >65 years old is over 21%. In Japan, the proportion of older adults is 26%. Accordingly, we have explained “super-aging society” in the introduction (page 3, lines 33-34).

Comment 6 from reviewer

In the introduction it is used the acronym ADL without its explanation.

Reply 6 to reviewer

We thank you for pointing out our insufficient description. We have spelled out ADL accordingly (activities of daily living) (page 3, line 48).

Comment 7 from reviewer

Regarding the diagnosis of LSS, which were the questions included in the self-administered questionnaire? It would be necessary to expand the information in this section.

Reply 7 to reviewer

Thank you for pointing out the lack of explanation about the questionnaire. We diagnosed LSS using a simple single sheet, self-administered, self-reported history questionnaire. It consisted of 15 questions associated with buttock and lower limb numbness or pain, and intermitted-claudication. Each question is scored from -1 to 5 points. We totaled the points of all questions and diagnosed LSS when the score was 13 points or greater. The sensitivity and specificity were 92.7% and 84.7% for diagnosing LSS in patients with lower limb symptoms due to LSS, arteriosclerosis obliterans, diabetes, or peripheral nerve diseases. These explanations have been added to the materials and methods (pages 5-6, lines 80-86).

Comment 8 from reviewer

It would be easier for the reader not using so many abbreviations. It is confusing and difficult to fully understand the paper in its current form.

Reply 8 to reviewer

We agree with your comment regarding not using too many abbreviations in this study. We used abbreviations; lumbar degenerative disease (LDD), lumbar spinal canal stenosis (LSS), mild cognitive impairment (MCI), quality of life (QOL), Japanese Orthopedic Association Back-Pain Evaluation Questionnaire (JOABPEQ), Mini Mental State Examination (MMSE), activities of daily living (ADL), visual analog scale (VAS), Kellgren-Lawrence grading (KL), diabetes (DM), and confidence interval (CI). We have reduced the number of abbreviations, other than the important key words in this study; LSS, MCI, QOL, JOABPEQ, MMSE, VAS and CI. We have reworded “LDD symptom” to “lumbar degenerative disease symptom.”

Comment 9 from reviewer

I would recommend to rewrite the Discussion section, as it repeats the main findings of the study but no proper discussion takes place.

Reply 9 to reviewer

We thank you for your valuable comment for improving the discussion of this study. We repeated the main findings of the study in the first paragraph, as well as in the middle of the second and third paragraphs and the conclusion; thus, we have deleted the first paragraph, which was the summary of the result. In the first paragraph, we discussed the prevalence of MCI and LSS combined. The comorbidity rate of MCI and LSS was not high (2.1%; however, the rate of MCI in participants with LSS　and the rate of LSS in participants with MCI were high (41%, 9.7%). According to a previous study, the risk factors of LSS presence, such as age or diabetes or low ankle-brachial index values, were similar to the risk factors of cognitive impairment. We have included this discussion in the first paragraph (pages 17-19, lines 220-257). In the second paragraph, we discussed that LSS presence and QOL decline due to low back pain were associated with MCI or MMSE summary score. It is clear that pain is related to cognitive impairment in the previous study. We suggested that a combination of LSS symptoms, such as pain or numbness of buttock or lower limbs or intermittent claudication, were related to cognitive impairment as with the previous study. It is clear that physical dysfunction associates with cognitive impairment. We suggest that physical dysfunction (limitation in range of lumbar motion) due to low back pain caused QOL decline and cognitive impairment. Several reports showed a causal relationship between pain or physical dysfunction and cognitive impairment; however, whether the intervention of pain or physical dysfunction improves cognitive impairment remains unclear. We have added that longitudinal study should be continued to clarify the causal relationship between lumbar degenerative disease and cognitive impairment (pages 19-21, lines 258-295).
